# Vitamin D Serum Levels in Subjects Tested for SARS-CoV-2: What Are the Differences among Acute, Healed, and Negative COVID-19 Patients? A Multicenter Real-Practice Study

**DOI:** 10.3390/nu13113932

**Published:** 2021-11-03

**Authors:** Luca Gallelli, Gaia Chiara Mannino, Filippo Luciani, Alessandro de Sire, Elettra Mancuso, Pietro Gangemi, Lucio Cosco, Giuseppe Monea, Carolina Averta, Pasquale Minchella, Manuela Colosimo, Lucia Muraca, Federico Longhini, Antonio Ammendolia, Francesco Andreozzi, Giovambattista De Sarro, G&P Working Group, Erika Cione

**Affiliations:** 1 Department of Health Science, School of Medicine, University of Catanzaro, Operative Unit of Clinical Pharmacology, Mater Domini University Hospital, 88100 Catanzaro, Italy; gallelli@unicz.it (L.G.); desarro@unicz.it (G.D.S.); 2 Department of Medical and Surgical Sciences, University Magna Graecia of Catanzaro, 88100 Catanzaro, Italy; gaiamannino@gmail.com (G.C.M.); elettramancuso@gmail.com (E.M.); giuseppe.monea93@gmail.com (G.M.); carolinaaverta90@gmail.com (C.A.); 3 Infectious Diseases Unit of Annunziata Hospital, 87100 Cosenza, Italy; filippoluciani@gmail.com; 4 Physical Medicine and Rehabilitation Unit, Department of Medical and Surgical Sciences, University of Catanzaro “Magna Graecia”, 88100 Catanzaro, Italy; ammendolia@unicz.it; 5 Operative Unit of Clinical Chemistry Laboratory, Pugliese Ciaccio Hospital, 88100 Catanzaro, Italy; pgangemi@aocz.it; 6 Department of Infectious Disease, Pugliese Ciaccio Hospital, 88100 Catanzaro, Italy; malattie.infettive@aocatanzaro.it; 7 Department of Microbiology and Virology, Pugliese Ciaccio Hospital, 88100 Catanzaro, Italy; minchellap@tin.it (P.M.); manuelacolosimo@hotmail.it (M.C.); 8 Department of General Medicine, ASP 7, 88100 Catanzaro, Italy; luciamuraca@alice.it; 9 Department of Anesthesiology and Reanimation, Pugliese Ciaccio Hospital, 88100 Catanzaro, Italy; flonghini@unicz.it; 10 Department of Pharmacy, Health and Nutritional Sciences, Department of Excellence 2018–2022, University of Calabria, 87036 Cosenza, Italy; erika.cione@unical.it

**Keywords:** vitamin D, COVID-19, SARS-CoV-2, gender difference, IL-6

## Abstract

Vitamin D might play a role in counteracting COVID-19, albeit strong evidence is still lacking in the literature. The present multicenter real-practice study aimed to evaluate the differences of 25(OH)D_3_ serum levels in adults tested for SARS-CoV-2 (acute COVID-19 patients, subjects healed from COVID-19, and non-infected ones) recruited over a 6-month period (March–September 2021). In a sample of 117 subjects, a statistically significant difference was found, with acute COVID-19 patients demonstrating the lowest levels of serum 25(OH)D_3_ (9.63 ± 8.70 ng/mL), significantly lower than values reported by no-COVID-19 patients (15.96 ± 5.99 ng/mL, *p* = 0.0091) and healed COVID-19 patients (11.52 ± 4.90 ng/mL, *p* > 0.05). Male gender across the three groups displayed unfluctuating 25(OH)D_3_ levels, hinting at an inability to ensure adequate levels of the active vitamin D3 form (1α,25(OH)2D3). As a secondary endpoint, we assessed the correlation between serum 25(OH)D_3_ levels and pro-inflammatory cytokine interleukin-6 (IL-6) in patients with extremely low serum 25(OH)D_3_ levels (<1 ng/mL) and in a subset supplemented with 1α,25(OH)_2_D_3_. Although patients with severe hypovitaminosis-D showed no significant increase in IL-6 levels, acute COVID-19 patients manifested high circulating IL-6 at admission (females = 127.64 ± 22.24 pg/mL, males = 139.28 ± 48.95 ng/mL) which dropped drastically after the administration of 1α,25(OH)_2_D_3_ (1.84 ± 0.77 pg/mL and 2.65 ± 0.92 ng/mL, respectively). Taken together, these findings suggest that an administration of 1α,25(OH)_2_D_3_ might be helpful for treating male patients with an acute COVID-19 infection. Further studies on rapid correction of vitamin D deficiency with fast acting metabolites are warranted in COVID-19 patients.

## 1. Introduction

The current pandemic infection of coronavirus disease 2019 (COVID-2019) is caused by severe acute respiratory syndrome coronavirus-2 (SARS-CoV-2), a novel RNA β-coronavirus that shares 79% sequence homology with SARS-CoV [1]. Even if to date there is no evidence that a specific therapy could improve the outcomes in patients with COVID-19, several pharmacological treatments, including vitamin D3, could negatively influence the infection progression acting on the immune system [1,2].

In fact, both immune dysfunction and cytokine storm are involved in the development of COVID-19 [3,4]; some authors have documented that vitamin D3 is able to improve the symptoms of SARS-CoV-2 infection because it acts on the immune system and modulates lung function [3], other authors have suggested that vitamin D3 supplementation may reduce the risk of SARS-CoV-2 infection [4,5].

Vitamin D3 (in animals, including humans), is considered biologically inactive until it undergoes two enzymatic hydroxylation reactions. The first takes place in the liver and it is mediated by the 25-hydroxylase, most likely cytochrome P450 CYP2R1, which forms 25-hydroxyvitamin D [25(OH)D_3_] (calcidiol, calcifediol) [6]. The second reaction takes place in the kidney and is mediated by the 1α-hydroxylase (also known as cytochrome P450 CYP27B1), which converts 25(OH)D_3_ to the biologically active hormone, 1α,25-dihydroxy vitamin D3 (1α,25(OH)_2_D_3_). The binding of 1α,25(OH)_2_D_3_ to the vitamin D receptor (VDR) induces the formation of a coactivator complex leading to target gene transactivation [7]. The VDR–RXRα coactivator complex can directly affect the genes to which VDR was previously attached, or it can reallocate and promote the transcription of other genomic sites, therefore leaving regulatory sequences uncovered for positive gene regulation by other transcription factors. This mechanism ensures the extra-skeletal effects of vitamin D3 on endothelial modulation, muscle mass and function, metabolic control, and the immune system, including cytokine expression profile [7,8,9,10,11].

Data from the UK-Biobank showed that although circulating vitamin D levels did not affect the risk of COVID-19 infection, the habitual use of vitamin D supplements was significantly associated with a 34% lower risk of COVID-19 infection (*p* = 0.034) [12]. Notably, vitamin D plays a physiologic role in regulating normal innate and adaptive immunity [13], and it has been reportedly shown to be able to suppress pro-inflammatory and stimulate anti-inflammatory cytokine formation, which might have a positive impact on COVID-19 patients [13,14]. In this scenario, COVID-19 pandemic has placed under the spotlight the correlation among IL-6, cytokine release syndrome, hyperinflammation state and COVID-19 fatality rate [15,16].

Our hypothesis is that there might be a strict correlation between vitamin D status and IL-6 serum levels. Nonetheless, albeit a role of vitamin D has been proposed in counteracting COVID-19 [3,4,13,14] through the potential modulation of the immune dysfunction and cytokine storm, to date, there is still a lack of evidence in the literature in terms of differences in serum levels across large cohorts, according to disease status.

Therefore, by the present study, we aimed to evaluate the levels of serum 25(OH)D_3_ in subjects tested for SARS-CoV-2 (acute COVID-19 patients, subjects healed from COVID-19, and non-infected ones). Furthermore, we sought to evaluate the effects of the supplementation of 1α,25(OH)_2_D_3_ on IL-6 and 25(OH)D_3_ serum levels in a sub-group of COVID-19 patients with chronic renal failure and hypovitaminosis D.

## 2. Materials and Methods

### 2.1. Data Collection

In this multicenter real practice study, we recruited patients with symptoms of an acute airway disease (e.g., cough, sore throat, breathing difficulties) referred to the Emergency Department of “Pugliese Ciaccio” Hospital, or to the Intensive Care Unit of the University Hospital “Azienda Policlinico Mater Domini”, both located in Catanzaro, Italy, over a 6-month period (from 30 March 2021 to 30 September 2021). All study participants underwent a naso-pharyngeal swab real-time reverse transcription polymerase chain reaction (RT-PCR) analysis for SARS-CoV-2. This study is part of the clinical trials recorded in clinicaltrial.gov (NCT04322513) and was conducted in compliance with the Institutional Review Board/Human Subjects Research Committee requirements. All participants, or their legal guardians, were asked to carefully read and sign an informed consent, and researchers provided to protect the privacy and the study procedures according to the Declaration of Helsinki and the Guidelines for Good Clinical Practice criteria, with pertinent National and International regulatory requirements. Data collection and reporting were performed in accordance with the Strengthening the Reporting of Observational studies in Epidemiology (STROBE) guidelines. The study was approved by the Institutional Ethics Committee (approval code: 2020.68).

### 2.2. Study Participants

Eligible patients were of both sexes, aged >18 years, with symptoms of an acute airway disease (e.g., cough, sore throat, breathing difficulties), with or without fever, muscle pain, or sudden anosmia or ageusia. A diagnosis of SARS-CoV-2 infection was postulated based on RT-PCR analysis of naso-pharyngeal swabs. The presence of anti-SARS-CoV-2 IgG was investigated to establish the existence of previous contacts with the virus. Patients who did not sign the informed consent or had been taking medicinal preparations and dietary supplements with vitamin D or cod-liver oil within the previous three months were not considered eligible for the study.

### 2.3. Experimental Protocol

After clinical, biochemical, microbiological, and radiological evaluation, patients were included and divided into four groups:

**Group 1**: SARS-CoV-2 acute infection with a positivity to naso-pharyngeal swab RT-PCR analysis (COVID-19);

**Group 2**: patients negative for both naso-pharyngeal swab RT-PCR and IgG anti SARS-CoV-2 analysis (no-COVID-19);

**Group 3**: patients healed from SARS-CoV-2 infection; with negative naso-pharyngeal swab RT-PCR analysis and positive to IgG anti SARS-CoV-2 (h-COVID-19);

**Group 4**: SARS-CoV-2 acute infection with a positivity to naso-pharyngeal swab RT-PCR analysis, and with hypovitaminosis D, who underwent 1-week treatment with 0.25 µg of 1α,25(OH)2D_3_ per day, at 1 week from the admission (vd-COVID-19).

### 2.4. Endpoints

The first endpoint was to determine a statistically significant difference in terms of serum 25(OH)D_3_ levels among SARS-CoV-2 healed patients (Group 3; h-COVID-19), SARS-CoV-2 infected patients (Group 1; COVID-19) and patients never infected by SARS-CoV-2 (Group 2; no-COVID-19). Moreover, the effects of 1α,25(OH)_2_D_3_ in COVID-19 infected patients (Group 4; vd-COVID-19) was evaluated.

The second endpoint was the correlation between serum 25(OH)D_3_ and IL-6 levels, in patients with extremely low serum 25(OH)D_3_ levels (<1 ng/mL).

Lastly, in all four groups, we also evaluated the gender-related statistically significant difference in serum 25(OH)D_3_ levels.

### 2.5. Reverse Transcription Polymerase Chain Reaction (RT-PCR) Assay for SARS-CoV-2

Naso-pharyngeal swab samples were collected for extracting SARS-CoV-2 RNA from patients suspected of having COVID-19. All test swabs were placed in a collection tube with 150 μL of virus preservation solution, and total RNA was extracted within 2 h using automated nucleic acids extraction and PCR setup (Nimbus, Seegene, Seoul, Korea). The suspension was used for RT-PCR assay of SARS-CoV-2 RNA. Two target genes for SARS-CoV-2, including RNA-dependent RNA polymerase and nucleo-capsid protein, were simultaneously amplified and tested during the RT-PCR assay. The RT-PCR assay was performed using a SARS-CoV-2 nucleic acid detection kit according to the manufacturer’s protocol (Allplex™ 2019-nCoV Assay—Seegene, Seoul, Korea). RT-PCR assay was performed under the following conditions: incubation at 50 °C for 15 min and 95 °C for 5 min, 45 cycles of denaturation at 94 °C for 15 s, and extending and collecting fluorescence signal at 55 °C for 45 s. A cycle threshold value (Ct-value) of less than 39 was defined as a positive test result, and a Ct-value of 40 or more was defined as a negative test. These diagnostic criteria were based on the recommendation by the Italian Health Institute according to World Health Organization (WHO) Berlin recommendation.

### 2.6. Serum 25(OH)D_3_ and Interleukin-6 (IL-6) Detection

Blood samples were collected from all the patients in the different groups at the time of hospitalization and then centrifuged. Serum aliquots were prepared and stored at −20 °C in screened and standard tubes for further analysis.

Serum samples were processed using chemiluminescent immunoassay technique through the Architect I1000^®^ (Abbott Diagnostics, Lake Forest, IL, USA; LOD 2.2 ng/mL), using an internal quality control system to ensure the assay validity. Each detection for 25(OH)D_3_ and IL-6 was performed in triplicate to reduce the technical error.

### 2.7. Statistical Analysis

Continuous data are expressed as mean ± standard deviation (SD) and categorical data as counts and percentages. A one-way ANOVA test was used to evaluate the differences within the groups. Differences identified by ANOVA were examined by using a Kruskal–Wallis test followed by Dunn’s multiple comparison test. The Pearson test was used to evaluate the correlation between serum 25(OH)D_3_ levels and IL-6 levels. Wilcoxon matched-paired singed rank test was employed to analyze the difference in 25(OH)D_3_ and IL-6 levels before and after treatment with 1α,25(OH)_2_D_3_ supplementation. GraphPad 5.0 software was used for the statistical analyses (GraphPad Software, San Diego, CA, USA).

## 3. Results

We recruited 133 patients of both sexes, and after clinical and laboratory evaluation, 16 were excluded (12.0 %), eight because their legal guardians did not sign the informed consent, and eight because they used vitamin D supplementation before the beginning of the study. The remaining 117 patients (87.9%) were included and assigned to the 4 study groups:

**Group 1:** COVID-19, *n* = 40 (30.1%): 27 males (67.5%) and 13 females (32.5%), with an age at diagnosis ranging from 38 to 83 years (mean 56.0 ± 7.7 years for males; 57.6 ± 13.7 years for females);

**Group 2:** no-COVID-19, *n* = 38 (28.5%): 23 males (60.5%) and 15 females (39.5%), with average age ranging from 25 to 72 years (mean 53.2 ± 9.5 years for males; 44.3 ± 13.2 years for females);

**Group 3:** h-COVID-19, *n* = 27 (20.3%): 19 males (70.4%) and 8 females (29.6%), with an age at diagnosis ranging from 21 to 63 years (mean 56.0 ± 7.7 years for males; 57.6 ± 13.7 years for females).

**Group 4:** vd-COVID-19, *n* = 12 (9.0%): 7 males (58.3%) and 5 females (41.6%), with an age at diagnosis ranging from 76 to 82 years (mean: 79 ± 2.2 years for males; 74.8 ± 2.9 years for females) with chronic renal failure, who underwent 1-week treatment with 1α,25(OH)_2_D_3,_ 0.25 µg per day.

Using the chemiluminescent immunoassay (CLIA) technique, we were able to identify subjects with insufficient serum 25(OH)D_3_ levels in respect to the actual range considered normal (>30 ng/mL).

**Figure 1:**
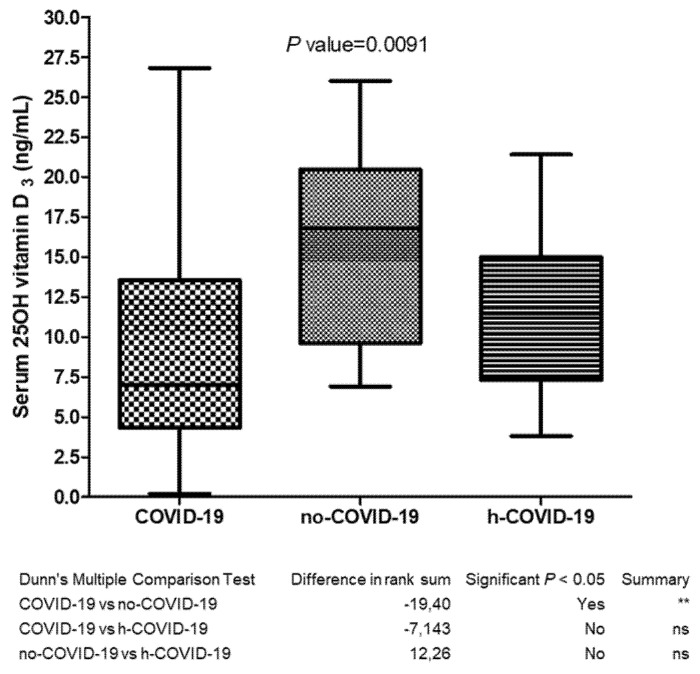
Serum 25(OH) vitamin D3 determination in COVID-19 (Group 1), no-COVID-19 (Group 2) and h-COVID (Group 3); ns = not significant, ** *p* value = 0.0091.

As per our primary endpoint, we evaluated the existence of a statistical difference in 25(OH)D_3_ serum levels among the three study groups. We documented that serum levels of 25(OH)D_3_ in COVID-19 patients (9.63 ± 8.70 ng/mL) were significantly lower (*p* = 0.0091) than values reported by no-COVID-19 patients (15.96 ± 5.99 ng/mL). Notably, we reported the absence of a significant difference (*p* > 0.05) between COVID-19 and h-COVID-19 patients (11.52 ± 4.90 ng/mL), albeit even in this case the COVID-19 group showed lower levels of serum 25(OH)D_3_ (see Figure 1 for further details).

**Figure 2:**
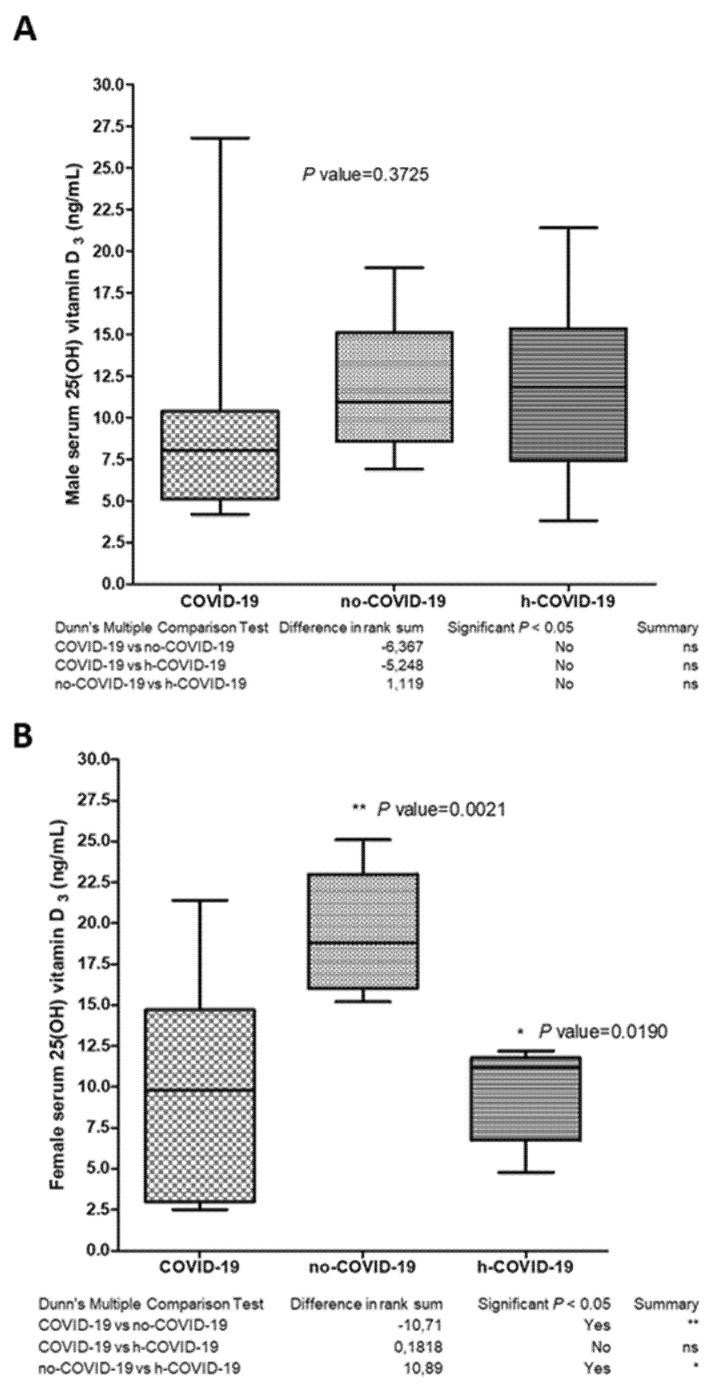
Serum 25(OH) vitamin D3 levels in COVID-19 (Group 1), no-COVID-19 (Group 2) and h-COVID (Group 3), dived per sex in (**A**) male and (**B**) female. ns = not significant. * *p* value = 0.0190, ** *p* value = 0.0021.

When we stratified the data by gender, we observed no difference in the male population, with a range of 25(OH)D_3_ serum levels of 10.4 ± 7.3 ng/mL in COVID-19 patients; 11.8 ± 4.3 ng/mL in no-COVID-19; and 11.7 ± 5.3 ng/mL in h-COVID-19 (Figure 2A).

On the other hand, we documented a significant difference in serum 25(OH)D_3_ levels in COVID-19 female patients (11.7 ± 9.5 ng/mL) in respect to no-COVID-19 group (17.7 ± 4.7 ng/mL) with a *p* = 0.006 (Figure 2B). The female no-COVID-19 group showed 25(OH)D_3_ levels significantly higher than the female h-COVID-19 (9.2 ± 2.8 ng/mL) group, with a *p*-value = 0.019 (Figure 2B). No significant differences emerged when we compared COVID-19 (11.7 ± 9.5 ng/mL) and h-COVID-19 (9.2 ± 2.8 ng/mL) female patients.

Overall, six patients belonging to Group 2 (COVID-19, four females and two males) showed extremely low serum 25(OH)D_3_ levels (<1 ng/mL). As per our secondary endpoint, we assessed circulating IL-6 levels in this subgroup of patients with extreme hypovitaminosis D (see Table 1 for further details).

Surprisingly, none of these patients exceeded circulating levels of IL-6, and they all stayed within the range of normality, with mean = 3.16 ± 1.2 pg/mL. Considering the small sample size (*n* = 6), we performed an exploratory non–parametric correlation analysis, which showed a positive correlation between 25(OH)D_3_ and IL-6 levels (Spearman correlation coefficient = 0.759, *p* = 0.04).

Besides that, the 12 patients in Group 4 (vd-COVID-19, 5 females and 7 males) showed low serum 25(OH)D_3_ levels (mean = 13.6 ± 1.1 ng/mL for females and 15 ± 3.5 ng/mL for males) at admission, with no difference by gender, consistent with the results obtained in Group 1. All of them were alive after 2 weeks from admission. As reported in Table 2 both the circulating levels of 25(OH)D_3_ and IL-6 significantly decreased after the administration of 1α,25(OH)_2_D_3_. IL-6 serum levels were high at admission in both females (127.64 ± 22.24 pg/mL) and males (139.28 ± 48.95 ng/mL), and dropped drastically after 1-week of supplementation with 1α,25(OH)_2_D_3_, showing mean IL-6 = 1.84 ± 0.77 pg/mL in females and 2.65 ± 0.92 ng/mL in males.

## 4. Discussion

In the present real-life study, we have evaluated the levels of serum 25(OH)D_3_ in recovered COVID-19 (h-COVID-19) patients, compared to acute infected (COVID-19) and non-infected ones (no-COVID-19).

Vitamin D acts as a lipophilic hormone and can be obtained from both nutritional intervention and sunlight exposure. Besides maintaining bone homeostasis, vitamin D is involved in the mechanisms of immune response [17]. Indeed, the scientific literature has recently pointed out the interaction between vitamin D and the immune system, suggesting that the former may play a key role in regulating normal innate and adaptive immunity [13]. In addition to this, previous studies have demonstrated an association between vitamin D deficiency and infectious processes [18,19,20]. Lu et al. [21], suggested that insufficient 25(OH)D_3_ levels are associated with an increase of both inflammatory cytokines and respiratory infections.

Notably, a retrospective study reported an association between low serum 25(OH)D_3_ levels and SARS-CoV-2 infection [22] and, more recently, other authors documented that insufficient serum 25(OH)D_3_ levels could represent a risk factor for the development of SARS-CoV-2 infection [5]. Accordingly, in this study, we documented the prevalence of 25(OH)D_3_ insufficiency (<30 ng/mL) in SARS-CoV-2 acute and healed infection as well as in Group 2, which encompassed patients without COVID-19 who had been admitted to the intensive care unit with symptoms of an acute airway disease. Of note, an insufficient level of 25(OH)D_3_ has been reported for several other conditions (e.g., COPD, obesity, diabetes, migraine, infections, cancer) [23,24,25,26,27,28,29]. Moreover, D’Avolio et al. [22] in 1377 control patients reported values of serum 25(OH)D_3_ levels lower than 30 ng/mL in patients with high levels of C reactive protein. In this concern, we found significantly higher serum 25(OH)D_3_ levels in no-COVID-19 patients compared to COVID-19 ones, but no differences between the former group and h-COVID-19.

Sexual bias is a hallmark in various diseases including infectious conditions [30], therefore, we decided to stratify our patients by gender, considering that males usually get the worst prognosis for COVID-19 [31]. In the present study, we highlighted a significant increase in serum 25(OH)D_3_ levels in the female no-COVID-19 group with respect to COVID-19 and h-COVID-19 female cohorts, whereas no differences were detectable among the male cohorts. The immune inflammatory response triggered by bacteria and virus infection is critical for the disease outcome, and the host benefits from an acute reaction when it is kept under tight control, with balanced protective vs. excessive/pathological inflammation. An example of viral infection models, known to cause more severe heart muscle inflammation in males than in females, is the Coxsackievirus B3 [32]. Notably, estradiol promotes immune regulation that favors a protective response in females [33]. Gender difference in vitamin D status is also a crucial research topic [34].

It has been recently reported that vitamin D deficiency is associated with increased levels of IL-6 in patients with HIV infections [35,36]. In agreement with this, experimental studies in mice revealed that vitamin D levels are inversely linked to IL-6 liver expression [37]. Low levels of serum 25(OH)D_3_ levels increase IL-6 synthesis and vice versa. In this context, targeting the IL-6 pathway could be an innovative therapeutic approach for COVID-19 patients, particularly in the elderly [15,38]. In our study, we noticed a subgroup of patients (four females and two males) characterized by extremely low levels of 25(OH)D_3_ (<1 ng/mL, tested twice for confirmation). Therefore, in this subpopulation we dosed circulating levels of IL-6 and, surprisingly, we found normal levels IL-6. Due to the small sample size, we cannot exclude the presence of confounding factors, such as a VDR mutation that could decreases the affinity of the receptor for its ligand and, therefore, increase the demand of 1α,25(OH)_2_D_3_ from extra renal tissues that increased 1α-hydroxylase expression in an inflammatory milieu [31]. On the other hand, it could be argued that in a scenario of chronic renal failure 25(OH)D_3_ is not efficiently converted to the active form 1α,25(OH)_2_D_3_, and patients have been reported displaying normal values of 25(OH)D_3_ levels (26 ng/mL) and hugely increased IL-6 concentrations (580 pg/mL). Clearly, in the presence of chronic renal failure any compensation provided by extra-renal calcitriol synthesis is insufficient to ensure adequate levels of the active vitamin D form. Furthermore, it should be noted that low serum levels of 25(OH)D_3_ are frequent in severe COVID-19 patients that commonly present several functional impairments [12,39,40,41,42].

When we corrected the condition of hypovitaminosis D in a small group of COVID-19 patients with chronic renal failure by supplementing 1α,25(OH)_2_D_3_ for one week, we observed a steep and significant decrease in circulating IL-6 levels. Our findings suggest that rapidly acting vitamin D metabolites are more likely to be effective in treating such patients than cholecalciferol itself. The emerging literature [41,42] supports this concept and it should be investigated in more depth by further studies on larger samples.

We are aware that the present study is not free from limitations: first, the small sample size that does not guarantee high external validity. Second, the absence of data on mutations of the VDR. Third, the fact that calcitriol is formed in many tissues including immune system cells, therefore its intracellular concentration may be higher than that found in the circulation, thus leading us to not assume that only those with chronic renal failure are short of calcitriol in their immune tissues.

However, this is the first study that: (i) evaluated patients in the real-life context, without exclusion for co-morbidities; (ii) investigated the levels of vitamin D in h-COVID-19; (iii) assessed the patients in a very short time and this excluded the possibility that sun exposition could modify 25(OH)D_3_ levels; (iv) performed a stratification by gender; (v) proved how calcitriol can be useful in COVID-19 patients with chronic renal failure.

## 5. Conclusions

Taken together, the findings of this multicenter cross-sectional study showed insufficient 25(OH)D_3_ serum levels in real-life patients with and without COVID-19, and in subjects healed from COVID-19. We collected evidences that males, who usually have a worst prognosis for COVID-19, display unfluctuating 25(OH)D_3_ levels that in turn could not ensure adequate levels of the active vitamin D form. Therefore, we could affirm that an administration of 1α,25(OH)_2_D_3_, without neglecting the hypercalcemic risk, might be considered as an adequate indication for treating male COVID-19 patients.

## Figures and Tables

**Table 1:** Serum levels of 25(OH)D_3_ and IL-6 determination in COVID-19 patients with extremely low 25(OH)D_3_ serum levels (*n* = 6).

Sex	Age (years)	25 (OH)D_3_ (ng/mL)	IL-6 (pg/mL)
Female	62	0.35	2.1
Female	63	0.25	2.6
Female	57	0.30	2.5
Female	60	0.72	2.8
Male	74	0.84	4.7
Male	69	0.90	4.3

**Table 2:** Serum levels of 25(OH)D_3_ (normal values >30 ng/mL) and IL-6 (normal values <6 pg/mL) determination after 1α,25(OH)_2_D_3_ administration in vd-COVID-19 patients (*n* = 12).

Sex	Age (Years)	25 (OH)D_3_ (ng/mL)			IL-6 (pg/mL)		
		Admission	After 2 Weeks	*p*-Value	Admission	After 2 Weeks	*p*-Value
F	73	15	13		132.1	2	
F	74	14	12		151.8	1.5	
F	73	13	11		111	1.8	
F	80	12	11		99	0.9	
F	74	14	13		144.3	3	
M	76	19	18	0.025	141	2	0.0022
M	82	12	11		200	3.5	
M	80	12	12		89	4.2	
M	81	13	13		65	3	
M	78	12	11		185	2.1	
M	77	18	17		162	2	
M	79	19	18		133	1.8	

Statistical analysis was performed using the Wilcoxon matched-paired singed rank test. M = male; F = female.

## Data Availability

Dataset is available on request.

## Appendix A

G&P Working Group enclosed: Davida Mirra (Department of Experimental Medicine L. Donatelli, Section of Pharmacology, School of Medicine, University of Campania Luigi Vanvitelli, Naples, Italy), Federica Pasceri and Ilenia Talotta (Department of Microbiology and Virology, Pugliese Ciaccio Hospital, Catanzaro, Italy.) Francesco Quintieri (Department of Infectious Disease, Pugliese Ciaccio Hospital, Catanzaro, Italy), Michela Carollo (Operative Unit of Clinical Chemistry Laboratory, Pugliese Ciaccio Hospital, Catanzaro, Italy), Antonio Siniscalchi (Department of Neurology, Annunziata Hospital, Cosenza, Italy), Eugenio Garofalo (Department of Medical and Surgical Science, Operative Unit of Anesthesiology and Reanimation School of Medicine, University of Catanzaro, Italy), Stefania Zampogna (Department of Children Diseases, Pugliese Ciaccio Hospital, Catanzaro, Italy), Girolamo Pelaia (Department of Health Science, School of Medicine, University of Catanzaro, Operative Unit of Respiratory Disease, Mater Domini University Hospital, Catanzaro, Italy), Alessia Fazio and Roberto Cannataro (Department of Pharmacy, Health and Nutritional Sciences, Department of Excellence 2018–2022, University of Calabria, Cosenza, Italy).
